# High-intensity gait training in subacute stroke resulted in increased discharge home from inpatient rehabilitation: a quality improvement study

**DOI:** 10.3389/fstro.2025.1681632

**Published:** 2025-11-17

**Authors:** Amanda Britton-Carpenter, Jade Thomas, Sandra A. Billinger

**Affiliations:** 1 Department of Rehabilitation, The University of Kansas Health System, Kansas City, KS, United States; 2 On With Life, Ankeny, IA, United States; 3 Department of Neurology, University of Kansas Medical Center, Kansas City, KS, United States

**Keywords:** high intensity gait training, subacute stroke, Stroke, stroke rehabilitation, discharge after stroke, home discharge

## Abstract

**Background:**

High-intensity gait training (HIGT) has emerged as a promising intervention to improve walking outcomes post-stroke. This quality improvement project aimed to increase the intensity of gait training for patients post-stroke in inpatient rehabilitation and evaluate HIGT's effects on functional mobility and discharge outcomes.

**Methods:**

Eighty-four patients post-stroke admitted to inpatient rehabilitation (2019–2021) were divided into HIGT (*n* = 32) and standard of care (SoC; *n* = 52) groups. Baseline characteristics were compared using *t*-tests or χ^2^ tests. Linear mixed-effects models evaluated changes in Inpatient Rehabilitation Facility Patient Assessment Instrument (IRF-PAI) (total and item-level) and secondary outcomes (6-min walk test (6 MWT), 10-Meter Walk Test (10 MWT), berg balance scale (BBS), Functional Gait Assessment, Five Times Sit to Stand Test, and Activities-Specific Balance Confidence Scale). Logistic regression examined predictors of discharge destination.

**Results:**

Groups were similar at baseline except for length of stay, which was longer for HIGT (16.6 vs. 11.1 days; *p* < 0.01). IRF-PAI Mobility improved significantly across all patients (*p* < 0.001), with a significant time × group interaction (*p* = 0.035) favoring HIGT. Item-level differences favored HIGT for “Chair/Bed-to-Chair Transfer” (*p* = 0.007), “Sit to Stand” (*p* = 0.005), and “Walk 10 Feet” (*p* = 0.008). Secondary outcomes improved within groups (*p* < 0.05) but did not differ significantly between groups. HIGT participants were more likely to discharge home (adjusted OR = 8.0, 95% CI [2.26–39.1], *p* = 0.003).

**Conclusion:**

Patients receiving HIGT demonstrated greater functional mobility gains and were more likely to discharge home than those receiving standard care. HIGT may enhance post-stroke recovery and support independent living. Further research should assess fidelity, long-term outcomes, and broader implementation.

## Introduction

The recovery of walking and discharge home are primary goals for stroke recovery during inpatient rehabilitation (IPR) (Gregory et al., 2010; Moore et al., 2022). Three treatment paradigms have traditionally been used as the basis of physical therapy intervention for individuals with stroke: impairment-based treatment, Bobath/Neurodevelopmental Treatment (NDT), and body-weight supported treadmill training (BWSTT) (Vaughan-Graham et al., 2015; Duncan et al., 2011; Veerbeek et al., 2014). For individuals post-stroke with a goal of improving gait speed and endurance, non-walking balance training, such as what occurs with impairment-based treatments, is not recommended as an intervention due to limited evidence (Hornby et al., 2020). Veerbeek et al. found NDT was less effective than other interventions for improving gait quality, gait speed, and length of stay (Veerbeek et al., 2014). While BWSTT does focus on walking training as the primary intervention, research findings did not support superiority when compared to a home exercise program for improving walking function post-stroke (Duncan et al., 2011).

In 2011, Hornby et al. published a review highlighting the potential implications of increased walking intensity for individuals post-stroke (Hornby et al., 2011). Since then, additional research has shown improved walking outcomes after implementing higher intensity walking (Holleran et al., 2014, 2015; Hornby et al., 2015, 2016; Boyne et al., 2016). High-intensity gait training (HIGT) has emerged as a promising treatment intervention for individuals with stroke and other neurologic conditions (Boyne et al., 2023, 2024; Moore et al., 2020). HIGT is variable-context stepping tasks performed at moderate-to-high aerobic intensities with an additional focus on increased repetition (Hornby et al., 2015). Variable-context stepping activities may include walking activities overground on even and uneven surfaces, treadmill walking, and stairs (Henderson et al., 2022b). HIGT is now a recommended treatment for individuals with chronic stroke who have a goal of improving their locomotion, but more research is needed for the efficacy of HIGT in subacute stroke (Hornby et al., 2020).

The goal of this quality improvement (QI) project was to increase the intensity of gait training for patients with stroke in IPR to improve their walking function. The purpose of this manuscript was to retrospectively analyze the potential changes in Inpatient Rehabilitation Facility Patient Assessment Instrument (IRF-PAI) Mobility Scores, outcome measures, length of stay, and discharge disposition to assess the effectiveness of the intervention.

## Materials and methods

### Project design and sample

For this quality improvement project, data was retrospectively collected on 84 patients with ischemic or hemorrhagic strokes admitted to The Acute Inpatient Rehabilitation Unit at the University of Kansas Health System in Kansas City, Kansas, within the same 3-month period (June, July, August) of 2019–2021. This project was submitted to the University of Kansas Medical Center Institutional Review Board for approval. The proposal did not require review as it was exempt due to the retrospective nature and goal to be a quality improvement project. The Acute Inpatient Rehabilitation Unit at the University of Kansas Health System is a 29-bed facility admitting ~130 patients post-stroke/year. All patients admitted to IPR received 3 h of rehabilitation 5 days/week spread across at least two disciplines; physical therapy (PT), occupational therapy (OT), and speech therapy. PT sessions were focused on improving functional mobility while OT sessions were focused on improving upper extremity function and independence with activities of daily living. Data were collected as part of standard PT services, which consisted of 30-, 45-, or 60-min sessions with daily variability to accommodate required services.

### Implementation

Prior to the initiation of the quality improvement project, two physical therapists working in the IPR chose to attend a continuing education course for HIGT for individuals post-stroke. After course completion, these two physical therapists conducted training on HIGT for the five additional IPR full-time physical therapy staff. This training included in-services, competency checkoffs, and mentoring sessions to ensure staff comfort and patient safety. Next, an education session was conducted with the unit's attending physicians regarding the intervention and to address potential safety concerns they may have. The physicians supported the physical therapists employing HIGT as an intervention and requested there be a discussion about the appropriateness of patients with unstable cardiac conditions such as arrhythmias to ensure patient safety.

As implementation of HIGT increased, staff had discussions regarding barriers and a primary barrier noted was a lack of necessary equipment, including enough heart rate monitors and gait training devices. As a result, our team applied for an internal grant which was funded to support HIGT within the health system. With the purchase of additional heart rate monitors and an overground gait training device, the team was able to more consistently implement HIGT.

### Intervention

Allocation of a patient to HIGT was the choice of the primary therapist. There was not specific inclusion or exclusion criteria for use of HIGT with a patient. If the therapist did not choose HIGT or the patient was not appropriate, the patient received standard of care (SoC) interventions, including gait training without focus on high intensity, transfer training, balance training, and strengthening. Patients in both groups received 60–90 min of physical therapy 5 days/week in order to be in compliance with insurance guidelines for IPR facilities. For patients in the HIGT group, not all physical therapy sessions focused on HIGT, due to factors such as family training, scheduling conflicts, or toileting needs.

HIGT interventions were variable walking activities, including overground walking (with or without body-weight support), treadmill walking (with or without body weight support), stairs, and obstacle navigation, such as uneven surfaces. Repeated transfer practice was not a specific focus of the intervention. Therapists provided appropriate levels of assistance to maintain safety while reaching an intensity level of 75–85% of age-predicted maximal heart rate for 45–60 min 5 to 7 days per week. The intensity and time targets were goals, not requirements for HIGT. Heart rate was monitored with a Polar OH10 monitor connected via Bluetooth to a department iPod. A physical therapy session was considered to be HIGT if the therapist monitored heart rate. Due to initial barriers within the documentation system, minutes spent in the target heart rate zone were not reported, and therefore, were not collected for this project.

### Outcomes

Outcome data was collected retrospectively from the patient's electronic medical record. All patients received scores from one (dependent) to six (independent) on the 15 IRF-PAI Mobility Score items at admission and discharge, with total possible scores ranging from 15–90 (Harmon and Sonagere, 2023; Services CfMM, 2024). Those items were as follows: roll left and right; sit to lying; lying to sitting on the side of the bed; sit to stand; chair/bed-to-chair transfer; toilet transfer; car transfer; walk ten feet; walk 50 feet with two turns; walk 150 feet; walking ten feet on uneven surfaces; one step (curb); four steps; 12 steps; and picking up an object from the floor. An IRF-PAI mobility change score was calculated by subtracting the admission mobility score from the discharge mobility score.

In addition to IRF-PAI data, the following information was collected from the electronic medical record: discharge location; length of stay; date of stroke; demographics; 6-min walk test (6MWT) assessed ambulation endurance; ten-meter walk test (10MWT) assessed gait velocity; Berg Balance Scale (BBS) assessed sitting and standing balance; Functional Gait Assessment (FGA) assessed dynamic balance; Five Times Sit to Stand Test (5 × STS) assessed functional lower extremity strength; and Activities-Specific Balance Confidence (ABC) Scale assessed balance confidence.

### Statistical analyses

Analyses were performed in IBM SPSS Version 29.0.0.0. Baseline demographic and clinical characteristics were compared between groups using independent-samples *t*-tests or Mann–Whitney *U* tests for continuous variables and χ^2^ tests for categorical variables. Linear mixed-effects models with random intercepts for participants were used to evaluate admission-to-discharge changes in IRF-PAI Mobility (total and item-level scores) and secondary outcomes (6MWT, 10MWT, BBS, FGA, ABC, 5 × STS), with fixed effects for time, group, and their interaction. Between-group differences in length of stay, age at admission, and days from stroke onset to admission were assessed with independent-samples *t*-tests.

## Results

### Patient characteristics

**Figure 1:**
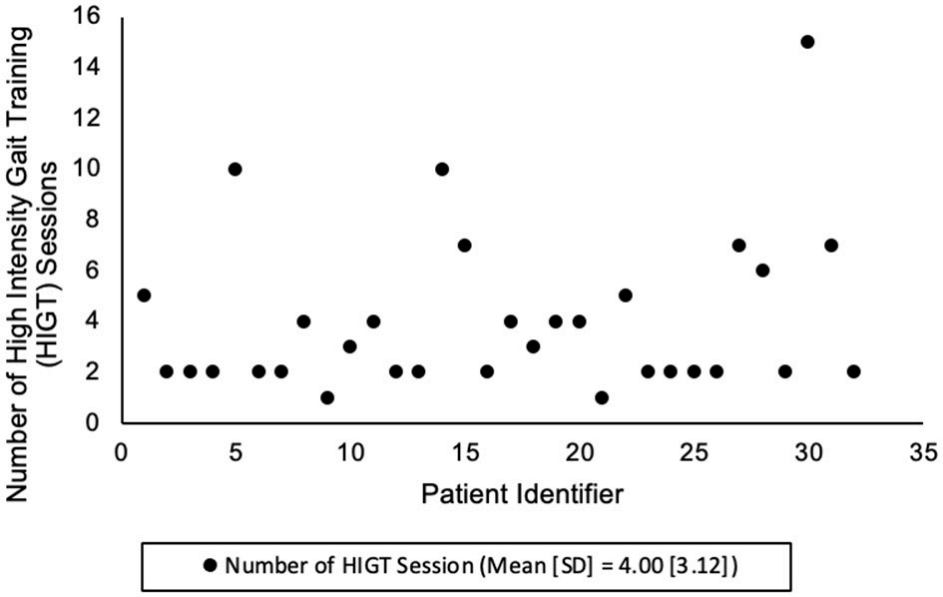
Number of HIGT sessions.

Eighty-four patients were included in this data set. Data regarding group distribution and group characteristics can be found in Table 1. There were no statistically significant differences between groups for any of the patient characteristics variables, with the exception of length of stay. The mean [SD] length of stay in days was 16.6 [7.7] for the HIGT group and 11.1 [6.58] for the SoC group (*p* < 0.01). The number of HIGT sessions performed with each patient can be found in Figure 1; the mean [SD] session was 4.00 [3.12]. No serious adverse events were the result of physical therapy intervention.

**Table 1:** Patient characteristics.

	**HIGT (*N* = 32)**	**SoC (*N* = 52)**	**Overall (*N* = 84)**
Group size, *n* (%)	32 (38.1%)	52 (61.9%)	84 (100%)
Age, mean [SD], years	67.3 [11.3]	68.8 [11.0]	68.21 [11.06]
**Sex**, ***n*** **(%)**			
Male	19 (59.4%)	33 (63.5%)	52 (61.9%)
Female	13 (40.6%)	19 (36.5%)	32 (38.1%)
**Race**, ***n*** **(%)**			
White	N/A	N/A	63 (75.0%)
Black	N/A	N/A	16 (19.0%)
Hispanic	N/A	N/A	3 (3.6%)
Asian	N/A	N/A	2 (2.4%)
Days from last known well to rehab admission, mean [SD]	9.8 [8.8]	10.6 [10.3]	10.3 [9.70]

Participant demographics and baseline characteristics for the high-intensity gait training (HIGT) and standard of care (SoC) groups. Values are presented as mean [SD] or n (%). No significant between-group differences were found for age, sex, or days from last known well to rehabilitation admission.

HIGT, high intensity gait training; SoC, standard of care.

### IRF-PAI scores

There were no significant differences in IRF-PAI total or item-level mobility scores between groups at admission, with the exceptions of “chair/bed-to-chair transfer” (*p* = 0.03) and “walk ten feet” (*p* = 0.05), suggesting similar baseline functional status; admission IRF-PAI data can be found in the Supplementary Materials. Across all patients, IRF-PAI Mobility scores improved significantly from admission to discharge (*p* < 0.001). The time × group interaction was significant for the IRF-PAI total score (*p* = 0.035), indicating that patients in the HIGT group demonstrated greater gains compared with SoC. The difference-in-differences for IRF-PAI Mobility score change is reported in Table 2. Item-level analyses revealed significant group differences in improvement for “Chair/Bed-to-Chair Transfer” (*p* = 0.007), “Sit to Stand” (*p* = 0.005), and “Walk 10 Feet” (*p* = 0.008). Other items improved within groups but without significant between-group differences; within group differences are reported in Table 3.

**Table 2:** Analysis of treatment effect on inpatient rehabilitation facility patient assessment instrument (IRF-PAI) GG mobility score.

	**HIGT (*N* = 32)**	**SoC (*N* = 52)**	**Overall (*N* = 84)**	***P*-value**
**Admission**				
Mean [SD]	32.9 [14.6]	36.0 [14.1]	34.79 [14.3]	0.32
**Discharge**				
Mean [SD]	62.3 [19.0]	56.1 [27.5]	58.5 [24.6]	0.26
**Change in Mobility Score from Admission to Discharge**				
Mean [95% CI]	29.4 [16.3]	20.2 [22.0]	23.7 [20.4]	0.044^*^

*p 0.05. Comparison of IRF-PAI total mobility scores at admission and discharge for patients receiving high-intensity gait training (HIGT) and standard of care (SoC). An IRF-PAI mobility change score was calculated by subtracting the admission mobility score from the discharge mobility score. Values are presented as mean [SD] unless otherwise indicated. The mean change from admission to discharge was significantly greater in the HIGT group compared to SoC (*p* = 0.044).

IRF-PAI, inpatient rehabilitation facility patient assessment instrument; HIGT, high intensity gait training; SoC, standard of care.

**Table 3:** Analysis of treatment effect and descriptive statistics for secondary outcomes.

**Outcome**	**Group**	**Missing admission data**	**Missing discharge data**	**Change**	**95% CI**	***P*-value**
Six-minute walk test (feet)	SoC	10	26	+92.7	[−4.9, 190.0]	0.062
	HIGT	2	8	+240.0	[115.0, 364.0]	< 0.001^***^
10-meter walk test (m/s)	SoC	11	5	−12.0	[−20.9, −3.2]	0.009^**^
	HIGT	24	11	−7.4	[−17.0, 2.2]	0.129
Berg balance scale	SoC	8	22	−22.1	[−40.8, −3.4]	0.021^*^
	HIGT	2	6	+0.47	[−23.4, 24.3]	0.969
Functional gait assessment	SoC	33	18	−19.6	[−39.4, 0.12]	0.051
	HIGT	23	34	−16.4	[−41.6, 8.7]	0.197
Five time sit to stand test (s)	SoC	24	12	+8.0	[−0.7, 16.7]	0.068
	HIGT	36	17	+4.5	[−7.0, 16.1]	0.428
Activities-specific balance confidence scale (%)	SoC	27	15	−16.0	[−36.9, 4.8]	0.130
	HIGT	36	23	−28.5	[−55.1, −1.9]	0.036^*^

^*^p < 0.05.

^**^p < 0.01.

^***^p < 0.001.

Within-group changes in secondary outcome measures from admission to discharge for patients receiving high-intensity gait training (HIGT) and standard of care (SoC). Values represent mean change with 95% confidence intervals (CI). Missing data reflect unavailable admission or discharge assessments. Positive change values indicate improvement except for the Five Times Sit to Stand Test, where a negative change indicates improvement.

HIGT, high intensity gait training; SoC, standard of care.

### Secondary outcomes

**Figure 2:**
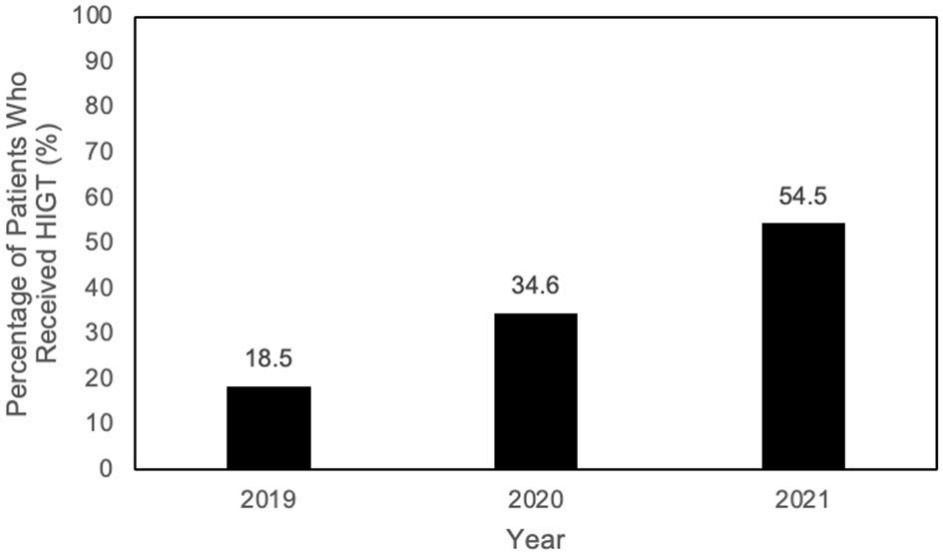
Increase in implementation of HIGT.

Secondary outcomes (6MWT, 10MWT, BBS, FGA, ABC, 5 × STS) showed significant within-group improvements (*p* < 0.05 for admission vs. discharge), though between-group differences were generally not significant. This data is featured in Table 3. Figure 2 demonstrates the increase of use of HIGT on the unit over a 3-year period.

### Discharge destination

**Figure 3:**
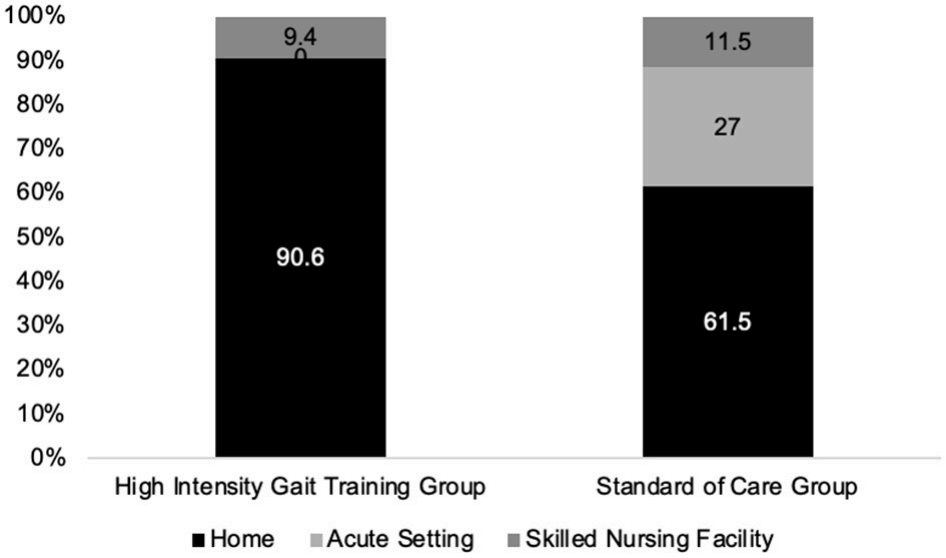
Discharge location.

In adjusted logistic regression, patients in the HIGT group were more likely to be discharged home compared with SoC, as depicted in Figure 3 (adjusted OR = 8.0, 95% CI [2.26, 39.1], *p* = 0.003). Higher admission IRF-PAI mobility scores were also associated with increased odds of home discharge (OR = 1.05 per point, 95% CI [1.01, 1.09], *p* = 0.033). Age, sex, and days from stroke to admission were not significant predictors.

## Discussion

The goals of this quality improvement project were to increase the usage of HIGT in one inpatient rehabilitation unit and to determine if HIGT improved outcomes for patients post-stroke. It was found that the HIGT group demonstrated a greater change in the IRF-PAI Mobility Score from admission to discharge, and they were discharged to a more independent setting as compared to conventional PT.

The overall discharge IRF-PAI Mobility Score was statistically different between groups. Even given the small number of sessions of HIGT, where we don't know fidelity to the target heart rate zone, those patients still had greater change. Interestingly, the HIGT group did not have statistically significantly higher scores on all individual IRF-PAI Mobility items, apart from “Chair/Bed-to-Chair Transfer,” “Sit to Stand,” and “Walk 10 Feet” items. IRF-PAI change scores are valid and reliable, and a higher IRF-PAI Mobility Score has been associated with increased independence and increased discharge home (Deutsch et al., 2023, 2022).

This project indicated patients with subacute stroke who participated in HIGT while in IPR had increased discharge home than patients who received conventional PT. A systematic review of patients without stroke admitted to an IPR demonstrated discharge home was strongly associated with being younger, married, racially and ethnically diverse, not experiencing depression, and having high functional and cognitive capabilities (Everink et al., 2016). Independence with transfers and walking short distances can be a barrier to discharge home as there is an increased caregiver burden. It is possible caregivers felt more comfortable with a home discharge given increased independence with those mobility items based on the IRF-PAI Mobility Score for this item in the HIGT group (Chevalley et al., 2023).

Patients who received HIGT had a longer length of stay than the patients who received standard care PT. This trend was also found by Moore et al. and thought to be due to better collaboration between the two levels of care involved in the study (Moore et al., 2020). A 2016 study found that increased severity of stroke was associated with a longer length of stay in IRF, and a longer length of stay was related to an increased likelihood of discharge home for those with severe impairments from their stroke (Camicia et al., 2016). While there were no significant differences in the overall admission or discharge IRF-PAI Mobility scores in this present study, the HIGT group did have a lower admission score. This could have contributed to the longer length of stay. Further, it is possible this occurred due to increased advocacy by therapists for these patients to stay longer in IPR due to the improvements they were making and their potential to be discharged home, instead of to a facility.

It should be noted that there was wide variability in the number of HIGT sessions received by patients. This was likely due to factors such as lack of equipment, including equipment to monitor heart rate and lack of comfort with the intervention, especially if the patient was admitted in the early phases of the implementation project. Another contributing factor was possibly system challenges that can occur in IPR settings, such as interruptions by other staff or the need for family training prior to discharge home. Similar barriers to implementation of HIGT were outlined by Mbalilaki et al. in 2024 (Mbalilaki et al., 2024).

Following the completion of this implementation project, it was reported that HIGT increased gait endurance more than conventional PT in IPR (Tapp et al., 2024). Another study found stepping activity, BBS scores, and paretic leg strength were predictors of walking outcomes in individuals with stroke participating in HIGT (Henderson et al., 2022a). Recent quality improvement and feasibility studies reported successful implementation of HIGT within IPR with individuals after subacute stroke (Moore et al., 2020; Henderson et al., 2022b). These studies demonstrated patients with stroke who received HIGT in IPR had greater improvements in walking speed, walking endurance, and balance (Moore et al., 2020; Henderson et al., 2022b). Improvements in 10 MWT, 6 MWT, and BBS were observed in this present project, but the statistical significance of these findings should be interpreted with caution due to the small sample size from missing data. A complete data set was not available for these variables, which is noted in Table 3.

## Limitations

Limitations of this study included a small sample size and missing data for the secondary outcome measures. This study also does not have information related to fidelity to the target heart rate zones. As group allocation was the choice of the primary therapist, the risk of selection bias was possible in this study.

## Conclusions

Implementation of HIGT in an IPR setting for individuals with subacute stroke was feasible and associated with greater improvements in IRF-PAI Mobility Scores and higher odds of discharge home compared to standard care. Although variability in the number and intensity of HIGT sessions occurred, patients demonstrated meaningful functional gains without increased safety concerns. These findings support the integration of HIGT into routine stroke rehabilitation practice and highlight the need for larger studies to confirm effectiveness and identify factors influencing response to this intervention.

## Data Availability

The raw data supporting the conclusions of this article will be made available by the authors, without undue reservation.
