# Supplementary material for: High-intensity gait training in subacute stroke resulted in increased discharge home from inpatient rehabilitation: a quality improvement study
Source: Front Stroke. 2025 Nov 17;4:1681632. doi: 10.3389/fstro.2025.1681632 (PMC12802622; doi:10.3389/fstro.2025.1681632)
Supplement: Supplementary file 1 [file Table_1.docx]

**Supplementary Table 1:** **Admission Data for Inpatient Rehabilitation Facility Patient Assessment Instrument**

Values are presented as mean [standard deviation] at admission for participants in the high-intensity gait training (HIGT) and standard-of-care (SoC) groups. P-values reflect between-group comparisons using independent-samples t-tests. Baseline mobility scores were comparable between groups across all items, except for the Chair to/from Bed transfer, which was significantly lower in the HIGT group (p = 0.03).

| **IRF-PAI Mobility Item** | **HIGT**  **N = 32** | **Standard**  **N = 52** | **Overall**  **N = 84** | **P-Value** |
| --- | --- | --- | --- | --- |
| **Roll Left and Right** | | | | |
| *Admission* | | | | |
| Mean [SD] | 3.47 [1.39] | 3.52 [1.51] | 3.50 [1.46] | 0.88 |
| **Sit to Lying** | | | | |
| *Admission* | | | | |
| Mean [SD] | 3.28 [1.37] | 3.38 [1.43] | 3.35 [1.4] | 0.75 |
| **Lying to Sitting** | | | | |
| *Admission* | | | | |
| Mean [SD] | 3.22 [1.36] | 3.27 [1.36] | 3.25 [1.35] | 0.87 |
| **Chair to/from Bed** | | | | |
| *Admission* | | | | |
| Mean [SD] | 2.41 [1.07] | 2.79 [1.11] | 2.64 [1.1] | 0.03 |
| **Toilet Transfer** | | | | |
| *Admission* | | | | |
| Mean [SD] | 2.44 [1.37] | 2.52 [1.29] | 2.49 [1.31] | 0.78 |
| **Car Transfer** | | | | |
| *Admission* | | | | |
| Mean [SD] | 1.31 [0.82] | 1.23 [0.67] | 1.26 [0.73] | 0.62 |
| **Walk 10 Feet** | | | | |
| *Admission* | | | | |
| Mean [SD] | 2.03 [1.15] | 2.58 [1.23] | 2.38 [1.2] | 0.05 |
| **Walk 50 Feet with Two Turns** | | | | |
| *Admission* | | | | |
| Mean [SD] | 2.06 [1.19] | 2.37 [1.24] | 2.25 [1.22] | 0.27 |
| **Walk 150 Feet** | | | | |
| *Admission* | | | | |
| Mean [SD] | 1.88 [1.18] | 2.21 [1.27] | 2.08 [1.24] | 0.23 |
| **Walk 10 Feet on Uneven Surface** | | | | |
| *Admission* | | | | |
| Mean [SD] | 1.59 [1.07] | 1.60 [1.03] | 1.60 [1.04] | 0.99 |
| **Curb Step** | | | | |
| *Admission* | | | | |
| Mean [SD] | 1.75 [1.11] | 1.92 [1.22] | 1.86 [1.17] | 0.52 |
| **Four Steps** | | | | |
| *Admission* | | | | |
| Mean [SD] | 1.81 [1.12] | 2.13 [1.31] | 2.01 [1.25] | 0.25 |
| **Twelve Steps** | | | | |
| *Admission* | | | | |
| Mean [SD] | 1.50 [0.98] | 1.60 [1.07] | 1.56 [1.03] | 0.68 |
| **Picking Up Object from Floor** | | | | |
| *Admission* | | | | |
| Mean [SD] | 1.88 [1.34] | 2.04 [1.40] | 1.98 [1.37] | 0.60 |

**Abbreviations: High Intensity Gait Training (HIGT); Standard of Care (SoC)**
